# A Home-Care Service for Frail Older Adults: Findings from a Quasi-Experiment in Milan

**DOI:** 10.1007/s12126-021-09462-6

**Published:** 2021-09-22

**Authors:** Simone Sarti, Francesco Molteni, Federica Cretazzo, Gianluca Giardini, Stefania Pozzati, Oriana Bruno

**Affiliations:** 1 Department of Social and Political Sciences, University of Milan, Via Conservatorio, Milan, Italy; 2 SOMET Ph.D. Programme – NASP, University of Turin, Turin, Italy; 3 Fondazione Sacra Famiglia, Milan, Italy

**Keywords:** Home-care, Mortality, Long-term care, Health services use, Milan (Italy)

## Abstract

Population aging is particularly pronounced in Italy. Recently, home-care services emerged as one of the desirable strategy when dealing with such situations of fragility. In this framework, we present the evaluation of a home-care service which was experimentally implemented by Fondazione Sacra Famiglia and Casa di Cura Ambrosiana in the 2017–2018 biennium. The service consisted of a twice-weekly nursing visit intended to monitor patient health conditions and to gather data constantly supervised by a geriatrician. The eligible population consisted of the users of San Carlo Hospital Emergency Department (91 individuals). Twenty of these individuals had access to the experimental home-care service. The results show the smaller probability of mortality for the treatment group compared to the control group, but similar probabilities in admissions to ER and in hospitalizations. These findings suggest that health home-care policies could reduce mortality by lessening the negative effects of relational isolation.

## Introduction

Population aging is a major topic in many industrialized countries. From a demographic point of view, it mainly reflects the combined effects of increasing life expectancy and a decreasing fertility rate (WHO, 2017). Even when compared with other industrialized countries, this phenomenon is particularly pronounced in Italy: in 2017 life expectancy at birth was 83.25 years and children for woman were 1.34 (source: World Bank).

The oldest-old (aged 85 or over) constitute a rapidly increasing segment of the population worldwide, but such longevity does not necessarily mean longer life expectancy in good health: the growing number of older persons with both health and social frailties clearly reflects this. Those who die around the age of 90 or over have a higher incidence of cognitive impairments like dementia, and functional limitations like difficulties with activities of daily living (ADLs), compared to those who die earlier (aged 85–89). Moreover, also multi-morbidity situations are very common in Italy as well as in other European countries and in the USA (Nagaratnam & Nagaratnam, 2019; Palladino et al., 2018). This is reflected also in population aging being associated with a declining life expectancy in good health of people aged 65 or more: from about 9 years in 2009 to 6–7 years in 2013, for both males and females (source: National Institute of Statistics, ISTAT—HFA).

As widely documented (see, for example, Santini et al., 2015, Holt-Lunstand et al., 2015), older people tend to maintain few (but essential) social relations and interactions if compared to younger persons. Such smaller and more homogenous social networks result in a progressive loss of a fundamental source of social and emotional support, also because of the limited contact frequency (Rafnsson et al., 2015; Tomini et al., 2016). Similarly to other well-documented clinical risk factors, this relational isolation of the older adults emerges as one of the main causes of morbidity and mortality (Tarugu et al., 2019). The problem is even more salient because the declining fertility rates come together with an increasing number of childless older people, if compared to the early twentieth century societies where only very few older persons lived alone.

For all these reasons, chronic debilitating diseases,—systemic diseases and, to a greater extent, mental illnesses like anxiety, depression, loneliness—and relational isolation of older adults are becoming increasingly common risk factors in the Italian population.

According to the WHO indications, all these questions invoke the expansion of long-term care (LTC) services, but the literature also depicts this as a complex and controversial issue mainly because of the enormous economic consequences in terms of nursing-home expenditures (Boland et al., 2017). In addition, LTC is shown to increase financial transfers, and to reduce (or even to prevent) paid employment for those who take care of older adults, such as family carers.

Within this debate, the location of care is a fundamental issue. Scholars (see, among many, Boland et al., 2017; Nagaratnam & Nagaratnam, 2019) argue that the large majority of older adults prefer to receive LTC at home, because it is a well-known physical and social environment. This mostly applies when care needs are moderate (Lehnert et al., 2019), while it becomes challenging when care needs are more intense. In addition, such home-care services are intended as means to reduce the adverse effects of potentially inappropriate medications and hospitalization, following model based on the IHI Triple Aim: better care, better health, and lower costs (Burgos-Diez et al., 2020). A topic of debate is the inappropriate use of medications (and their sub-use in particular) by older adults at home (Wauters et al., 2016). More broadly, in a recent meta-review, Luker and colleagues (Luker et al., 2019) argue that preventative home visits do not avoid or delay residential aged care admissions. Health service usage outcomes (such as hospital admissions and emergency department attendance) do not seem to be influenced by interventions at home.

Concerning this, a literature review about home-care services by Tourigny and colleagues (2015) finds that home-care interventions often included comprehensive geriatric assessments (CGAs) and follow-up visits. For example, some of them include preventive home visits (PHV) programs to improve quality of life and reduce risk of mortality in old people. Common feature behind such interventions is that they are based on a “multidimensional medical, functional, psychosocial, and environmental evaluation of their problems and resources.” (Bouman et al., 2008: 2).

In general, their findings suggest that PHV might have the potential to reduce mortality, especially of the youngest-old (aged 80 or less) and, if in combination with CGAs, clinical evaluations and follow-up visits, to also increase functional autonomy. Concerning this, Luker et al. (2019) stress that detailed information on interventions that enable people to remain in their own home is scarce, and the existence of a variety of interventions does not allow simple comparative analysis. Authors note that further studies should be undertaken and more detailed descriptions of the intervention programs should be made to better understand the pros and cons of remaining at home for frail older adults.

On the basis of all these considerations, it becomes clear why national and international research attributes increasing importance to the study of policies intended to improve the life conditions of older adults through home-care interventions. The present paper is placed within this theoretical framework, with the main objective to present and describe a particular experience of a home-care service. In particular, we want to assess an experimental home-care service (named *Virgilio 2.0*) dedicated at reducing the inappropriate use of health services while maintaining a good level of care and assistance for vulnerable older persons. We evaluated three outcomes: mortality, hospitalization and access to ER.

In the first section (THE RESEARCH CONTEXT) the context in which the research took place will be described. Then, a section will be devoted to presenting the data and the methods used (DATA AND METHODS). Finally, the results will be reported (RESULTS) and discussed (DISCUSSION).

## The Research Context

This paper presents the results of an experimental home-care service implemented by Fondazione Sacra Famiglia ONLUS (FSF) and Casa di Cura Ambrosiana (CCA) in the 2017–2018 biennium. FSF is the institution responsible for many social and health services (extended care nursing homes, integrated home-care assistance, day-care centres, retirement houses, etc.) as well as for the rehabilitation ones (Italian law 833/78, ex art. 26) distributed among 17 different centres in Lombardy, Piedmont, and Liguria. This structure relies on almost 2,000 employees working with almost 1,600 guests in the residential care services, and almost 400 employees in the semi-residential ones. Conversely, CCA offers health-care services assimilable to ordinary admissions, day surgery, specialized, general and geriatric rehabilitation, and out-patient care. Overall, it manages 132 beds and employs almost 300 healthcare professionals.

In the 2017/2018 biennium, these two institutions implemented a home-care service which consisted of constantly monitoring the patients’ health conditions, and of gathering data on such conditions. At the same time, a geriatrician was responsible for taking the appropriate decisions according to the collected data (therapy change, hospitalization, etc.).

In 2018, FSF and CCA commissioned to the University of Milan a consulting activity whose main aim was to assess this service. This home care service, named “Virgilio^1^ 2.0”, was intended to mainly reduce the inappropriate use of health services (in particular hospitalization and emergency departments) while maintaining at the same time a good level of care and assistance. More specifically, the Virgilio 2.0 service was financed by the Cenci Gallingani Foundation ONLUS with the aim of enhancing home-care practices through the use of remote assistance and remote-control technologies. Accordingly, the main objective was to maintain older adults at home, also in the case of periodic checks on their health conditions and constant contact with a network of assistants.

Moving to the organization of this home-care service, the patient was followed by doctors (geriatricians), nurses and social care workers (e.g. social workers, socio-health personnel, etc.) all belonging to the FSF and CCA. In order to integrate health-care services with those of social support, the involvement of the social services of the municipality of residence was also possible.

The first step of the project consisted in a doctor, usually a specialist in geriatrics, defining the intensity of the intervention on the basis of the medical history. Then, each patient was followed by a case manager, either a nurse or a social worker according to the predominant need. The doctor and the case manager were responsible for coordinating the monitoring activity of the patients, and the possible interventions. Based on the evaluation carried out by the doctor, the patients were visited at home by nurses or socio-health personnel (“OSS” in Italy) 2 or 3 times per week. The nurses (or the OSSs) were equipped with a backpack containing the instruments necessary to measure blood glucose, saturation, body weight circumference, temperature and pressure, spirometry, and to conduct electrocardiogram examinations (further details on this equipment can be found in Appendix A). The data collected by means of the equipment were transmitted via tablet to a web platform that recorded information and fed the personal file of each patient. Professional health workers constantly read and interpreted such data during their working days. When necessary, these data were also used to modify the pharmacological therapy in order to reduce risks of exacerbation, deterioration or improper use of the emergency department. As regards the experimental phase described in this study, the home care service was free of charge.

## Methods

The population eligible for the Virgilio 2.0 service consisted of a subgroup of 91 individuals coming from the San Carlo Hospital Emergency Department. This subgroup was determined by the hospital staff according to the following characteristics: being aged 70 + , social-health frailty (need of extra-hospitalized care, presence of at least two morbidities and a therapy with five or more active principles), and residential proximity to the hospital. Twenty patients had access to the experimental home-care service between July and November 2017. Although there were no selection biases in sampling these 20 users, no pure randomized sampling was performed. This resulted in a quasi-experimental design because the assignment of the medical intervention was not at the discretion of the investigators. All patients authorized the treatment, scheduling a domiciliary visit to receive the study documents (the Patient Information Sheet). The informed consent was signed by the patients themselves or, in case of cognitive impairment, by their caregivers.

The characteristics of the sample fit with the use of propensity score matching (PSM) models in order to determine the effect of the treatment (participation in the Virgilio 2.0 program) on the health outcomes of the patients (Guo & Fraser, 2015). The main aim of PSM models is to reduce the bias due to confounding variables which is likely to be found when trying to estimate the treatment effect by simply comparing outcomes. This technique works by "pairing" subjects with similar characteristics coming from the treated and the control group, in order to estimate the average effect of the treatment (ATE—Average Treatment Effect). The ATE estimates represent the difference of treatment, on average, on the outcomes Y_1_ and Y_0_, where Y_1_ represents the state of health taken as reference and Y_0_ the alternative state. More precisely, the ATE can be defined as the average effect that would be observed if everyone, treated or not, had received treatment, compared to the situation in which no one had received treatment. This procedure represents a step further toward a more robust causal explanation (Li, 2013).

The preliminary step necessary in order to apply these models is to identify a list of potential confounders. Such a list was drawn from two sets of information. Looking at the set of basic socio-demographic data, the choice was to control for age (from 72 to 95 years), gender (male, female) and cohabitation condition—as a proxy for relational support (not known, with children, with caregiver, with spouse, alone). Basic descriptive information about these variables is provided in Table 1.

**Table 1:** Basic socio-demographic variables used for matching

	**SAN CARLO**		**VIRGILIO**	
	N	Average	N	Average
**GENDER**				
Female	49	/	10	/
Male	22	/	10	/
**RELATIONAL SUPPORT**				
Unknown	7	/	6	/
Living with children/relatives	14	/	3	/
Living with professional caregiver	8	/	1	/
Living with partner	21	/	5	/
Living Alone	21	/	5	/
**AGE**	71	86.0	20	82.8
**TOTAL**	**71**	**/**	**20**	**/**

In addition to this basic information, it was also possible to access results from many clinical tests administered to the patients before the beginning of the programme. An overview of the tests and of the results is provided in Table 2. Among them, it has been acknowledged that there were no significant differences for many of the tests (Braden, Brass, Pfeiffer, MNA): the similarity of the measures is a good indication that such characteristics do not represent potential confounders. On the contrary, the choice was taken to introduce into the PSM models the results of the tests for which significant differences between the treated and the control group were observed. In fact, what emerge are the best average performances of the users involved in the Virgilio 2.0 program as regards the 4AT, MORSE and Barthel tests. The Barthel test measures the functional autonomy of the subjects, the MORSE test evaluates the mobility and the 4AT the potential cognitive deficit and delirium. To be noted is that these tests were administered during the admission to the San Carlo Hospital, thus before both the selection of the treatment group and the start of the program.

**Table 2:** Tests results and keys for interpretation (in bold differences statistically significant at 95%, t test)

	**SAN CARLO**		**VIRGILIO**			
	N	Average	N	Average	Range	Thresholds for interpretation
BRADEN	73	15	16	16.5	6–23	< 9: very high risk, > 19 no risk
Brass	70	20.4	19	19.1	0–40	> 20: discharge with support
Mini Nutritional Ass	54	10.2	17	10.7	0–30	> 24: Normal state. < 17: Bad nutritional state
Pfeiffer	52	4.5	19	3.4	0–10	The higher the results, the better the performance
**4AT**	**62**	**3.3**	**18**	**1.4**	**0–12**	**1–3: possible cognitive decline. 4 + : possible delirium**
**MORSE**	**73**	**40.6**	**14**	**31.8**	**0–125**	**The higher the result, the higher the risk of falls**
**Barthel**	**65**	**30.3**	**20**	**51.5**	**0–100**	**The higher the result, the better the autonomy**

In the context of the assessment of the project, the main outcome evaluated was the mortality of the patients. Information on mortality outcomes were considered at one year after the admission to the San Carlo Hospital, from July to November 2018. These data come from FSF, which gave us administrative information about mortality, hospitalizations and ER admissions of the patients. In order to supplement the information about mortality, also the number of hospitalizations and ER admissions have been evaluated. In particular, six objective indicators were taken into account:

- **Mortality I:** deceased vs. not deceased (listwise): the missing cases were due to missed compilations or unavailability of the MORSE, Barthel or AT4 cards at the hospital admission.
- **Mortality II:** deceased vs. not deceased (replacement of missing values with averages): missing values ​​on the three variables MORSE, Barthel and 4AT have been replaced with group means.^2^
- **Number of hospitalizations I:** from 0 to 5 (listwise).
- **Number of hospitalizations II:** from 0 to 5 (considering only not deceased).
- **Number of ER admissions I:** Not (0) or Yes (1) (listwise).
- **Number of ER admissions II:** Not (0) or Yes (1) (considering only not deceased).

## Results

Table 3 presents the raw results stemming from the administrative data collection at one year after the admission to the San Carlo Hospital. Effects are estimated in difference in probabilities. The probability to still be alive is higher for Virgilio patients (+ 0.28) and the difference is statistically significant (Chi square Test). Meanwhile, probabilities in hospitalization and in ER admissions are slightly lower for Virgilio users (-0.07 in both cases), although these differences are not statistically significant.

**Table 3:** Raw results of the quasi-experimental design, administrative data

	**SAN CARLO**	**VIRGILIO**	**TOTAL**	**VIRGILIO** **effect** **(difference in probabilities)**	**P-value** **(Chi-square test)**
**Live/death**					
**1 Not deceased**	44	18	62	0.280 (1 vs 2)	0.017
**2 Deceased**	27	2	29		
**Total**	71	20	91		
**ER admission**					
**1 Not**	39	9	48	-0.075 (1 vs 2)	0.557
**2 Yes**	32	10	42		
**Total**	71	19*	90		
**Hospitalizations**					
**0**	43	10	53	-0.079 (0 vs 1 +)	0.097
**1**	14	4	18		
**2**	8	2	10		
**3 + **	6	3	9		
**Total**	71	19*	90		

^*^In one case data is missing

**Table 4:** Propensity Score Matching models to evaluate Virgilio 2.0 on mortality, hospitalizations and ER admissions *outcomes* (ATE estimates, standard errors, p-values, confidence intervals 95% and number of cases considered)

		**Measure**	**ATE**	**AI robust std err**	**Prob**	**C.I. 95%**		**Valid cases**
						**Low**	**Upp**	
	**INDICATORS**							
**1**	Mortality I	0/1	-0.307	0.063	0.000	-0.432	-0.183	65
**2**	Mortality II	0/1	-0.274	0.067	0.000	-0.407	-0.142	91*
**3**	Hospitalizations I	0–5	0.323	0.371	0.385	-0.405	1.051	65
**4**	Hospitalizations II	0–5	0.166	0.386	0.667	-0.591	0.924	48
**5**	ER admissions I	0/1	0.123	0.254	0.628	-0.374	0.620	65
**6**	ER admissions II	0/1	-0.166	0.195	0.394	-0.549	0.216	48

^*^All cases considered

Notwithstanding the interest of these preliminary results, we have previously stressed the risk for these effects to be potentially affected by selection biases. Therefore, we adopted a more sophisticated statistical model (PSM) with the aim to alleviate such biases.

Given that the reference group consisted of the Virgilio 2.0 home care service users, the average treatment effect (ATE) values have to be interpreted as the effects of the treatment. Since mortality is the reference category, negative ATE values ​​should be read as health protective factors. In other words, ATE values ​​below zero mean a decrease in the risk of incurring the corresponding situation (mortality) if a person is subject to treatment (participation in Virgilio 2.0), while positive values ​​should be read in the opposite direction. The uncertainty concerning the estimates is indicated by the AI robust standard error (for details see Abadie & Imbens, 2011) and, similarly, by the confidence intervals. As usual, if the confidence interval does not involve zero, the effect should be considered as statistically significant.

The ATE value for the first mortality indicator is -0.307. Since it is a dichotomous indicator (deceased or not deceased), this value can be interpreted in favour of Virgilio 2.0. In fact, being involved in the program results in a lower probability (30.7%) of dying. This estimate is statistically significant with a significance level of 0.01 (the asymptotic probability is 0.000). Quite coherently, the second indicator has an ATE of -0.274, which means again a lower probability (27.4%) of dying. Despite different assumptions on missing data, results are consistent between the two indicators.

As a robustness check, we also computed the average treatment effect on treated (ATET) value, which is an additional estimate generally used to compare the effect of a treatment in a propensity score matching model. At the end of the home-care service, a smaller probability of mortality around 23% ATET (C.I. 95%) for the treatment group was observed, compared to the control group (27% ATET, C.I. 95%). These results together provide robust evidence in favour of the positive effect of the Virgilio 2.0 in reducing mortality of older adults. In addition, it should be noted that 0.280, that is 28%, in percentage, was the raw measure of the effect (see Table 3).

As far as ER admissions and hospitalizations concern, results of the analysis show that all the ATEs estimates are not statistical significant. Confidence intervals are large and contain zero. This means that that are not substantial difference in health service access between VIRGILIO and SAN CARLO users. (Table 4)

## Discussion

For what concerns an overall assessment of this study, some limitations cannot be ruled out. Firstly, the size of the sample, which was possible to test, is rather small. Secondly, a not perfectly randomized selection of the treatment group was performed. However, the use of propensity score matching models enabled us to strongly reduce possible biases due to these selection problems.

Despite these limitations, the findings provide evidence that public health policies of home-care services and a continuous monitoring of the psychological and physical conditions of older adults (using specialised personnel equipped with remote control technologies) might reduce mortality risk. This main result is coherent with the strand of literature which stresses the positive role of home-care services in increasing chances to remain living at home (Luker et al., 2019; Tourigny et al., 2015).

At the same time, results show that hospitalizations and ER admissions are not influenced by the home care service. The aforementioned meta-review of Luker and colleagues (2019) has showed similar findings in 11 different studies (but not in other 4): despite heterogeneity in the research designs and potential methodological bias (as lack of blinding participation to the trial and control groups), home interventions do not seem to influence health service usage.

At one year from this home-care service, the risk of mortality was lower for participants to Virgilio 2.0 than for those who did not participate, but the use of traditional healthcare services was not. This may be due to an effect we can label ‘just-in-time health care’. Although this does not decrease the potential problems (and therefore the use of traditional healthcare), monitoring patients at home could help them using health service in a more timely way and to reduce risk of mortality. This result integrates the findings showing that health-care services based on professional home visits allow to better answer to the basic care needs of the older adults. (Burgos-Dìez et al., 2020).

However, in order to overcome some of the weaknesses linked to the experimental context, further research is needed to corroborate this empirical result. This is rather urgent because in Italy, as in other industrialized countries, the rate of old and very old people living in relational isolation conditions will probably reach epidemic proportions by 2030 and LTC programs will become increasingly important.

Moreover, these problems could be intensified by the recent COVID-19 outbreak. Scientific evidence on COVID-19 identifies older adults as the social category being exposed to the highest risks due to the pandemic. What followed were health prevention policies suggesting isolating them socially, and this generates major concerns among aging scholars (Armitage & Nellums, 2020).

For this reason, this study appears to be pioneering for further experiments on how policies can better fulfil the increasing and heterogenous demand for long-term care of the older population (https://www.who.int/aging/long-term-care/en/).

## Footnotes

1 Virgilio, Virgil in English, was Dante's companion and guide in the Divine Comedy.

2 In the case of MCAR—Missing Completely At Random, and when missing values ​​are attributable to purely random effects, the substitution with group means is considered a good approximation. The MCAR test by Little (1988) on the three variables considered gives a positive result: Chi-square = 13.276, DF = 8, Sign. = 0.103. The hypothesis of randomness of missing values ​​is therefore accepted (or rather, the hypothesis of non-random origin is rejected).
